# Retraction: Light-triggered nitric oxide release and targeted fluorescence imaging in tumor cells developed from folic acid-graft-carboxymethyl chitosan nanospheres

**DOI:** 10.1039/d0ra90103b

**Published:** 2020-10-19

**Authors:** Laura Fisher

**Affiliations:** Royal Society of Chemistry Thomas Graham House, Science Park, Milton Road Cambridge CB4 0WF UK advances-rsc@rsc.org

## Abstract

Retraction of ‘Light-triggered nitric oxide release and targeted fluorescence imaging in tumor cells developed from folic acid-graft-carboxymethyl chitosan nanospheres’ by Rijun Gui *et al.*, *RSC Adv.*, 2014, **4**, 30129–30136, DOI: 10.1039/C4RA03034F.

The Royal Society of Chemistry hereby wholly retracts this *RSC Advances* article due to concerns with the reliability of the data in the published article.

The TEM image in Fig. 2a duplicates data in a number of other publications, but reported as different materials.^1–5^

The TEM image in Fig. 2c duplicates data in other publications, but representing different materials.^4,6^ However, an additional particle has been copy and pasted into Fig. 2c of this *RSC Advances* article, which indicates that this image has been manipulated.

Given the number and significance of the concerns about the validity of the data, the findings presented in this paper are no longer reliable.

Rijun Gui opposes the decision to retract. Ajun Wan, Yalei Zhang, Huili Li and Tingting Zhao were contacted but did not respond.

Signed: Laura Fisher, Executive Editor, *RSC Advances*

Date: 21^st^ September 2020

## Supplementary Material

## References

[cit1] Gui R., An X. (2013). RSC Adv..

[cit2] Xu Z., Wu Z., Sun J., Gui R. (2015). Mater. Chem. Phys..

[cit3] Gui R., Jin H., Liu X., Wang Z., Zhang F., Xia J., Yang M., Bi S. (2014). Chem. Commun..

[cit4] Gui R., Wan A., Liu X., Jin H. (2014). Chem. Commun..

[cit5] Jin H., Gui R., Sun J., Wang Y. (2018). Talanta.

[cit6] Gui R., Wang Y., Sun J. (2014). Colloids Surf., B.

